# Implementation of the Community-based Health Planning and Services (CHPS) in rural and urban Ghana: a history and systematic review of what works, for whom and why

**DOI:** 10.3389/fpubh.2023.1105495

**Published:** 2023-06-26

**Authors:** Helen Elsey, Mary Abboah-Offei, Aishwarya Lakshmi Vidyasagaran, Dominic Anaseba, Lauren Wallace, Adanna Nwameme, Akosua Gyasi, Andrews Ayim, Adelaide Ansah-Ofei, Nina Amedzro, Delanyo Dovlo, Erasmus Agongo, Koku Awoonor-Williams, Irene Agyepong

**Affiliations:** ^1^Department of Health Sciences, Hull York Medical School, University of York, York, United Kingdom; ^2^School of Health and Life Sciences, University of the West of Scotland (London Campus), London, United Kingdom; ^3^Faculty of Public Health, Ghana College of Physicians and Surgeons, Accra, Ghana; ^4^Dodowa Health Research Centre, Research and Development Division, Ghana Health Service, Accra, Ghana; ^5^Department of Social and Behavioural Sciences, School of Public Health, University of Ghana, Accra, Ghana; ^6^School of Nursing and Midwifery, University of Ghana, Accra, Ghana; ^7^Ghana Health Service, Accra, Ghana

**Keywords:** Community-based Health Planning and Services (CHPS), health services administration and management, primary care, public health, social medicine, urban health

## Abstract

**Background:**

Despite renewed emphasis on strengthening primary health care globally, the sector remains under-resourced across sub–Saharan Africa. Community-based Health Planning and Services (CHPS) has been the foundation of Ghana's primary care system for over two decades using a combination of community-based health nurses, volunteers and community engagement to deliver universal access to basic curative care, health promotion and prevention. This review aimed to understand the impacts and implementation lessons of the CHPS programme.

**Methods:**

We conducted a mixed-methods review in line with PRISMA guidance using a results-based convergent design where quantitative and qualitative findings are synthesized separately, then brought together in a final synthesis. Embase, Medline, PsycINFO, Scopus, and Web of Science were searched using pre-defined search terms. We included all primary studies of any design and used the RE-AIM framework to organize and present the findings to understand the different impacts and implementation lessons of the CHPS programme.

**Results:**

*N* = 58 out of *n* = 117 full text studies retrieved met the inclusion criteria, of which *n* = 28 were quantitative, *n* = 27 were qualitative studies and *n* = 3 were mixed methods. The geographical spread of studies highlighted uneven distribution, with the majority conducted in the Upper East Region. The CHPS programme is built on a significant body of evidence and has been found effective in reducing under-5 mortality, particularly for the poorest and least educated, increasing use and acceptance of family planning and reduction in fertility. The presence of a CHPS zone in addition to a health facility resulted in increased odds of skilled birth attendant care by 56%. Factors influencing effective implementation included trust, community engagement and motivation of community nurses through salaries, career progression, training and respect. Particular challenges to implementation were found in remote rural and urban contexts.

**Conclusions:**

The clear specification of CHPS combined with a conducive national policy environment has aided scale-up. Strengthened health financing strategies, review of service provision to prepare and respond to pandemics, prevalence of non-communicable diseases and adaptation to changing community contexts, particularly urbanization, are required for successful delivery and future scale-up of CHPS.

**Systematic review registration:**

https://www.crd.york.ac.uk/prospero/display_record.php?RecordID=214006, identifier: CRD42020214006.

## 1. Introduction

Globally there is a renewed interest and emphasis on strengthening primary health care (1, 2). Yet, across sub–Saharan Africa, primary health care is under-resourced, and attention directed to prestigious central referral hospitals and vertical programmes (3). There are few examples of national strategies for delivery of primary and community prevention and care that have developed from context-specific research to identify the most effective approach. The Community-based Health Planning and Services (CHPS), which has been national policy in Ghana since 1999, is one such example (4). CHPS delivers community level health promotion, prevention and primary clinical care in Ghana's multi-tiered primary health care system, to provide the appropriate health services to communities, whilst supported by a system of referrals to higher levels of care when needed (5). The wealth of quantitative and qualitative assessments of CHPS over three decades provide valuable insights into the successes and challenges of the programme (6). Learning and sharing these lessons is important not only for similar resource-constrained countries across sub-Saharan Africa but is vital to inform adaptations to the CHPS programme in Ghana itself, particularly at a time of epidemiological and demographic transition. Ghana, like all countries in sub-Saharan Africa is experiencing rapid urbanization with an urban growth rate of 4.2 and 65% of the population is expected to be urban by 2030 (7). This is coupled with a rising prevalence of non-communicable disease whilst still contending with infectious diseases (8).

While there are still challenges in resourcing primary care within rural Ghana, within-urban analysis highlights the inequities in health outcomes, particularly for children aged under 5 years (9). This highlights the need to improve the accessibility and quality of prevention and primary care services for urban poor communities, the majority of whom are dependent on often unregulated, private providers (10).

**Figure 1:**
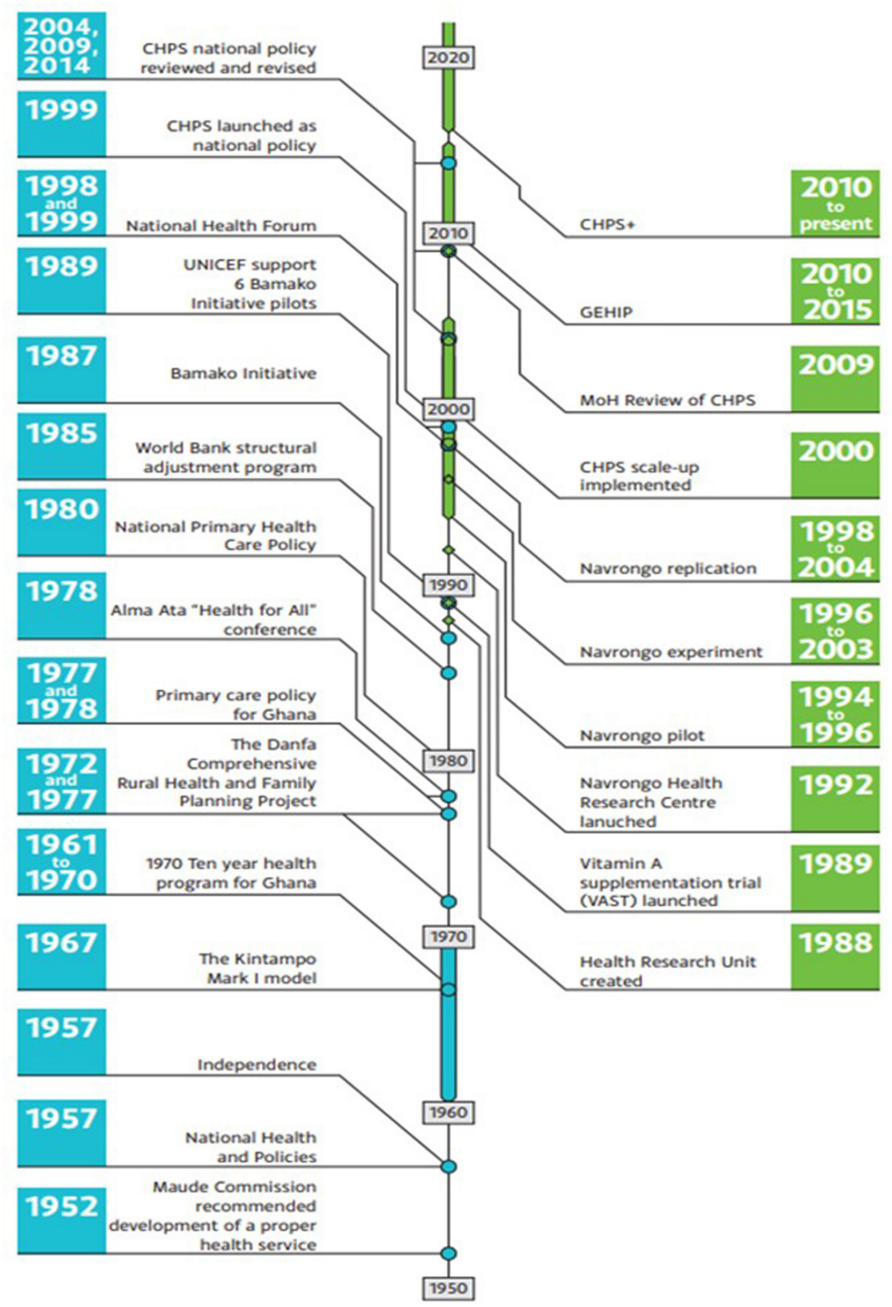
The history of CHPS from its inception.

The evolution of the CHPS programme in Ghana occurred out of progressive national and health system learning over several decades, with policy makers drawing on lived and research evidence from these processes. Figure 1 shows key health policy development milestones in blue, and the development of CHPS in green. Five years before independence, the Maude Commission of 1952 recommended health service development focusing on hospitals and health centers resulting in an increase from 89 doctors and three health centers in 1952 to 141 doctors and 46 health centers by 1961 (11). The following 10-year health programme (1961–1970) emphasized an efficient rural health service with integration of hospitals and health centers, training of medics and paramedics and intersectoral collaboration to tackle the social determinants of health (12). Concerns however remained over the slow pace of trickle-down of benefits to communities. Initiatives to reach rural communities followed with the 1967 Kintampo Mark I model of “cottage hospitals” and health posts (13) followed by The Danfa Comprehensive Rural Health and Family Planning Project (1972–1977) which developed a new cadre of community-based workers known as Health Education Assistants (HEA) to better reach rural communities. Evaluations showed that the HEA approach improved adoption of family planning but struggled to bring about changes in health practices when other support services were not available (14–16). To address this the 1977/78 primary care policy emphasized community involvement with the selection and training of village health workers, and the introduction of Village Development Committees to stimulate intersectoral collaboration (13, 17). Tiers from national through regional, to district, sub-district and community were developed. Later in 1978, 134 member states approved the WHO declaration of Alma-Ata and the translation of the declaration into action resulted in a plethora of uncoordinated initiatives at community level with much emphasis on volunteerism and local support for community health workers (CHW).

Ghana, like many other countries in sub-Sharan Africa, was in a period of economic decline and stress throughout the 1970 and 1980's, and in 1985 started a World Bank structural adjustment programme that involved significant out-of-pocket user fees at point of service across the health sector. The results were catastrophic for the poorest, many of whom lived in rural areas in a country that though progressively urbanizing, was still predominantly rural (18). Senior policy makers were keen to reduce reliance on user fees and community volunteers and find ways to enable patients' financial protection, address health systems weakness particularly at sub-district and community levels (19) and coordinate donor programmes.

The adoption of the Bamako Initiative under the leadership of UNICEF in 1987 presented an opportunity to address these challenges. The initiative aimed to increase availability of healthcare services at community level, with essential drugs supplied by donors slightly above cost-price with profits sustaining future provision (20–23). Despite initial skepticism from senior policy makers, the Ministry of Health (MoH) in Ghana began implementation in six pilot districts in 1989. The district health director and team developed a structured programme for selection, training, support, and supervision of volunteer community health workers who would be paid by medicine sales. At this time rural areas were the focus as the most deprivation and need were found here. The internal evaluation in 1992 highlighted the limitations of relying on volunteers with *ad hoc* payment mechanisms based on medicine sales. There was a realization that community-based health workers integrated within the formal health system, receiving a regular salary and with formal community health nurse training were more likely to achieve success. This learning paved the way for the Navrongo Community Health and Family Planning Project (CHFP) where existing cadres of community health nurses were redeployed from health centers and health posts to live and work in the community, with responsibility for a wider catchment population. Senior policy makers, understanding the value of robust evaluation, ensured research became an integral part of the design, implementation and evaluation of CHFP, which became known as the “Navrongo Experiment.” Following the initial 1994 pilot, the programme was launched in 1996 with a focus on bringing essential health services closer to the communities, with particular emphasis on hard-to-reach rural areas (5). Initial strategies involved retraining and deploying health staff to communities, utilizing traditional institutions and support structures to organize and mobilize communities, and providing “doorstep” services such as preventive care, family planning, and immunization services (24). This combination of health staff deployment with community volunteer mobilization became the recommended “Navrongo model.” Results demonstrated that the strategies were both feasible and improved the primary health care impact, particularly around child mortality and fertility indicators (24–26). Construction of a compound in each community was found to be essential, not only as a base for outreach and provision of primary care services, but to provide accommodation for the community health nurse. Within this rural context, land was abundant and willingly provided by communities. Following a successful replication of the strategies in Nkwanta in 1998, CHPS was declared a national policy in 1999, with roll-out throughout Ghana from 2000 onward, using Navrongo and then Nkwanta as exemplars to inform scale up (5).

### 1.1. Components of CHPS: 15 steps and milestones

**Figure 2:**
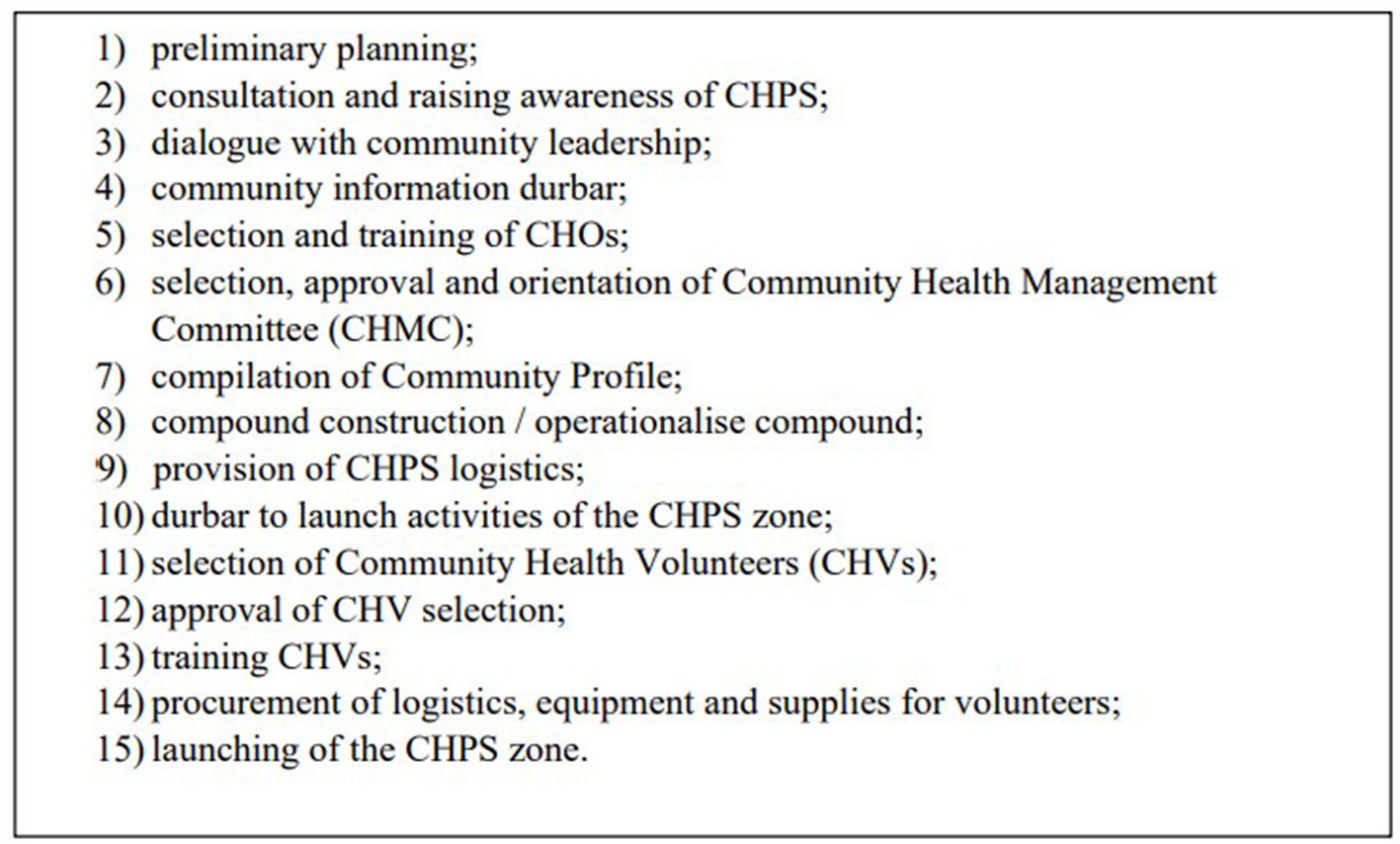
The 15 steps to CHPS implementation.

Today, the key characteristics of the early Navrongo and Nkwanta pilots remain, with community-based care provided by a resident professional nurse known as a Community Health Officer (CHO) supported by community volunteers, as opposed to conventional facility-based and “outreach” services. A key strategy for the successful introduction of CHPS in a community is close engagement with the traditional leaders to ensure commitment to the CHPS concept. This aims to trigger further community participation and mobilization of volunteers, first to construct a CHPS compound and then to support implementation of health services. The process has been detailed in a series of 15 steps to guide successful CHPS implementation (6, 27) (see Figure 2). Services provided by the CHOs include household visits for antenatal care, family planning services, and health education; outreach clinics, providing child welfare services; and school health services. In-service training workshops organized for CHOs serve to improve basic clinical and midwifery services and develop diplomacy, communication, and counseling techniques (6).

The careful evidence-based design, clearly specified features of CHPS and national roll-out make it a prime subject for continued evaluation, as can be seen by the many quantitative and qualitative studies conducted since its inception. Learning the lessons from these evaluations is vital if primary health care is to develop and respond to the changing context within Ghana and beyond. In light of this, we aimed to understand the challenges and facilitators to the implementation of the CHPS programme and its impact on health and process outcomes. To do this we conducted a systematic review of published and unpublished empirical studies of the CHPS programme to address the following objectives:

1. To describe the effectiveness of the CHPS programme in improving health and health service outcomes.
2. To identify the extent to which CHPS has been able to reach different population groups and geographical settings, both rural and urban.
3. To describe the facilitators and barriers to implementation of the CHPS programme and the maintenance of this implementation over time.

The systematic review was performed according to the Preferred Reporting Items for Systematic Reviews and Meta-Analysis (PRISMA) statement (28).

## 2. Methods

### 2.1. Protocol and registration

The protocol for the review was registered on the PROSPERO International prospective register for systematic reviews (CRD42020214006).

### 2.2. Review design

We conducted a systematic review of published and unpublished empirical studies on the CHPS programme in both rural and urban areas in Ghana. To understand not only which outcomes CHPS improves, but also for whom, in what context and why, we conducted a mixed-method systematic review using a results-based convergent design where the quantitative and qualitative findings are synthesized separately and then brought together in a final narrative synthesis (29). This allowed us to collate quantitative results on the outcomes of CHPS and qualitative, mixed-methods or quantitative results on the mechanisms (e.g., health system, participant, or contextual factors) that may influence effectiveness.

### 2.3. Inclusion and exclusion criteria

We included all primary studies of any design from both published and unpublished literature that reported CHPS implementation and evaluation in rural and urban Ghana. Quantitative, qualitative and mixed methods studies that evaluated CHPS spanning from 1994 (launch of the Navrongo experiment, forerunner to CHPS) to March 2022 were eligible. See Supplementary Table 2 for detailed description of the inclusion and exclusion criteria.

### 2.4. Search strategy and terms

An electronic search was planned on EMBASE (Ovid), MEDLINE (Ovid), PsycINFO (Ovid), Web of Science, and Scopus and included studies from database inception up to October 2020, to identify relevant published and gray literature on CHPS implementation in Ghana. An updated search was conducted in March 2022, using variants of the search terms associated with “Community-based health planning and services” and “Ghana” and “CHPS implementation” and “health outcomes” (see Supplementary Table 1). Both index terms and free texts were incorporated into the search strategy to make our search as sensitive as possible. We searched the reference lists of included studies, national CHPS annual reports from Ghana Health Service (GHS), GHS policy, planning monitoring and evaluation reports, and unpublished theses from the School of Public Health of the University of Ghana. We drew heavily on the knowledge of co-authors with long experience of CHPS to develop a list of organizational websites to search for evaluations including: USAID (United States Agency for International Development), UNFPA (United Nations Population Fund), JICA (Japan International Cooperation Agency), DfID now FCDO (Department for International Development), The Doris Duke Charitable Foundation, Columbia University; Royal Netherlands Embassy; GIZ (Deutsche Gesellschaft fur Internationale Zusammenarbeit), KOICA (Korea International Cooperation Agency), KOFIH (Korea Foundation for International Healthcare), WHO (World Health Organization), and CHAG (Christian Health Association of Ghana).

### 2.5. Data screening and extraction

One reviewer (MA-O) conducted an initial screening of titles and abstracts to remove any studies not conducted in Ghana. The remaining titles and abstracts of all identified studies were screened by two reviewers. Screening was organized using Rayyan software (https://www.rayyan.ai/). Where insufficient information was available in the abstract, full texts of papers were independently assessed by two reviewers and any uncertainty resolved by a third reviewer. Data extraction was performed independently by two reviewers using a standardized proforma, with any discrepancies resolved by a third reviewer. Variables extracted include: Authors/year, Region/District of study (classify as urban or rural), aims/objectives, study design and methods, target population, quantitative results and measures of health outcomes (e.g., child mortality, fertility, and maternal mortality) and any proximal outcomes (e.g., uptake of services, satisfaction, availability of providers, and community involvement). Qualitative themes were also extracted from findings and discussion sections.

### 2.6. Quality assessment

As this review included all primary studies of any design, a number of quality assessment tools designed for specific study types were implored in assessing the quality of included studies. Among them were The Cochrane risk of bias tool (30), used to assess the quality of randomized controlled trials (RCTs); ROBINS-I was used to assess risk of bias in non-randomized intervention studies (31); and the risk of rigor (32) within qualitative studies was assessed using the Critical Appraisal Skills Programme (CASP) Qualitative Research Checklist (33), see Table 1 for included studies and corresponding quality scores.

**Table 1:** Included studies with quality score.

**References**	**Focus/research question**	**Region and urban/rural**	**Study design and Sample**	**Quality**
**Quantitative studies—plausibility trials, chronologically (*****n*** = **8)**				
Debpuur et al. (26)	Impact of the initial 3 years of CHFP on contraception and fertility	Upper East, Rural	8,998 women (15–49 years)	High
Phillips et al. (34)	Demographic and health impact of CHFP with a view to scaling up results	Upper East, Rural	139,000 individuals	High
Binka et al. (35)	Demographic and health impact of CHFP with a view to scaling up results	Upper East, Rural	139,000 individuals	High
Pence et al. (24)	Impact of CHFP on under-5 mortality during 1993–2000	Upper East, Rural	52,801 children and 52,801 mothers	High
Phillips et al. (36)	Long-term impact of CHFP on fertility	Upper East, Rural	47,036 women (15–49 years)	Medium
Bawah et al. (37)	Contribution of CHPS to mitigate effects of poverty on childhood mortality	Upper East, Rural	94,599 under-five children	High
Bawah et al. (37)	Effect of GEHIP on under-5 mortality and associated factors	Upper East, Rural	7,044 under-five children and 5,914 women	High
Asuming et al. (38)	Family planning and unmet need impact of GEHIP	Upper East, Rural	5,914 women (15−49 years)	High
**Quantitative studies—other designs, chronologically (*****n*** = **19)**				
Awoonor-Williams et al. (39)	Exposure to CHPS and change in health-seeking behavior and health knowledge	Oti, Rural	Cross-sectional, 831 women (15–49 years)	Low
JICA (40)	Project for the scaling up of CHPS implementation in region	Upper West, Rural	Secondary data/programme evaluation, NA	Low
Naariyong et al. (41)	Comparing technical process quality of ANC between CHPS and non-CHPS areas	Eastern, Rural	Cross-sectional, 600 mothers (15–49 years)	Medium
Aikins et al. (42)	Evaluation of Facilitative Supervision Visits (FSV) component of CHPS	Upper West, Rural	Secondary data analysis, NA	Medium
Wood and Esena (43)	Community utilization of CHPS	Central, Rural	Cross-sectional, 175 heads of households	Medium
Johnson et al. (44)	Impact of CHPS on the uptake of skilled birth care	National, Rural, and Urban	Secondary data analysis, 4,349 births between 2003 and 2008	High
Awoonor-Williams et al. (45)	Monitoring systems to gauge CHPS coverage in all GEHIP districts	Upper East, Rural	Analysis of routine health service data	Low
Ferrer et al. (46)	HBC and CHPS implementation on utilization, treatment and satisfaction	Multiple, Rural	Cross-sectional, 1,356 carers of children under-5	Medium
Ferrer et al. (47)	Effectiveness of iCCM and CHPS on disease knowledge and health behavior	Multiple, Rural	Cross-sectional, 1,356 carers of children under-5	Medium
Ferrer et al. (48)	Cost-effectiveness of iCCM and CHPS on diagnosis and treatment of under-5s	Multiple, Rural	Cross-sectional, 1,356 carers of children under-5	Medium
Wiru et al. (49)	Patronage of CHPS, factors associated with their use and challenges faced	Bono East, Rural	Cross-sectional, 171 community members	Medium
Sakeah et al. (50)	Role of CHPS in women having PNC visits and factors associated	North East, Rural	Cross-sectional, 650 women who had delivered in the past 5 years	Medium
USAID (51)	Quality and relevance of pre-service and in-service education of CHPS workers	Multiple, Rural, and Urban	Cross-sectional, 401 majority CHNs, followed by enrolled nurses, midwives	Low
Braimah et al. (52)	Contribution of CHPS policy to women's access to PHC services	Upper West, Rural	Cross-sectional, 805 women	Medium
GHS (53)	Verification exercise to determine the functionality of all CHPS zones	National, Rural, and Urban	Cross-sectional, NA	High
Maly et al. (54)	Access and quality of CHPS services after 2–4 years of project support	Western, Rural	Post-test, non-equivalent control design, 426 community members	Medium
Amponsah et al. (55)	Process evaluation on MCHNP and possible barriers to implementation	Eastern, Urban	Cross-sectional, NA	Medium
Kweku et al. (56)	Relevance of community involvement and community perception of CHPS	Volta, Rural	Cross-sectional, 1,008 community members	Medium
Kweku et al. (57)	Community utilization and satisfaction with CHPS services	Volta, Rural	Cross-sectional, 1,008 community members	Medium
**Qualitative studies, chronologically (*****n*** = **26)**				
Nyonator et al. (58)	Qualitative Systems Appraisal (QSA) of why CHPS is implemented in some districts, but stalled in others	Volta, Rural	Qualitative diagnostic approach, using focus group (19) with district managers, sub-district health teams, clinic and community-based nurses, community leaders, men and women of reproductive age	High
Binka et al. (59)	Independent, in-depth assessment of CHPS progress	Upper East, Rural	Qualitative, using desk review, in-depth and key informant interview, field visit	High
Ntsua et al. (60)	Diagnostic appraisal of delivering family planning services using CHPS model	National, Rural, and Urban	Qualitative, using desk review, in-depth and key informant interview and focus group with CHOs, women (15–49 years) and men in partnerships	High
Adongo et al. (61)	Male involvement in family planning in communities with and without CHPS	Multiple, Rural	Qualitative descriptive, using in-depth interview (62) with CHOs, CHVs and health managers; focus group (12) with male and female community members	High
Awoonor-Williams et al. (63)	Lessons learned from CHPS scaling up in region where the pace has been much more rapid than other regions	Upper East, Rural	Desk review of reports and qualitative interviews with district and regional directors	Low
Baatiema et al. (64)	Assessing participatory process in CHPS	Upper West, Rural	Spider-gram, using in-depth interview (17), focus group (2) and community conversation with service users, providers, community health committee members	High
Adongo et al. (61)	Implementation challenges and lessons from introducing rural CHPS experiences to an urban environment	Greater Accra, Urban	Analysis of routine health service data (mainly women 15–49 years)	Low
Krumholz et al. (65)	Facilitating and constraining factors in CHPS scaling up	Upper East, Rural	Qualitative, using in-depth interview (12) with key managerial staff current CHPS system managers	High
Sakeah et al. (66)	Extent to which CHO midwifery program is integrated into CHPS	Upper East, Rural	Case study, using in-depth interview (67) with CHO-midwives, supervisors, District Directors, heads of maternity wards, tutors of midwifery schools, health professionals, community leaders and residents	High
Sakeah et al. (68)	Extent of community participation in CHPS skilled delivery program	Upper East, Rural	Case study, using in-depth interview (12) with CHO-midwives	High
Atuoye et al. (69)	Transportation barriers to access maternal and child health services	Upper West, Rural	Qualitative, using focus group (2) with male and female participants, aged 18–70 years	High
Dalaba et al. (70)	Effect of CHPS on reproductive preferences and contraceptive use	Upper East, Rural	Qualitative, using in-depth interview (5) with community chiefs and elders and focus group (8 male and 8 female panels)	High
Bougangue and Ling (62)	Male involvement in various aspects of maternal health care	Central, Rural	Qualitative, using in-depth interview and focus group with married men, CHOs, CHVs, and community leaders	High
Assan et al. (71)	Barriers and facilitators of CHPS through a systems-centric perspective	Multiple, Rural, and Urban	Qualitative, using in-depth interview (41) with national, regional, district, and sub-district/local participants	High
Atinga et al. (72)	How and why women and children are disadvantaged in CHPS implementation	Upper West, Rural, and Urban	Case study, using focus groups (5) with community informants, in-depth interview with clients (71), and staff (13)	High
Nwameme et al. (73)	Reactions of health care personnel on implementation of CHPS in Accra	Greater Accra, Urban	Qualitative, using in-depth interview (19) with CHPS staff and officials	High
USAID (74)	Formative research to adapt the CHPS model to urban settings	Multiple, Urban	Unclear	Medium
Woods et al. (75)	Contribution of CHPS to community health sustainability	Upper West, Rural	Qualitative, using in-depth interview and focus group	High
Yakubu (76)	Factors (health service delivery, socio-cultural, economic) influencing utilization of CHPS	Northern, Rural	Qualitative, using in-depth (25) and key-informant (5) interview and focus group (12) with community members and key informants	High
Amoah (77)	State and functioning of CHPS from a social capital perspective	Ashanti, Rural	Qualitative, using in-depth interview (11) and focus group (2) with younger and older adults	High
Assan et al. (67)	Challenges to achieving UHC through CHPS	Multiple, Rural and Urban	Qualitative, using in-depth interview (41) with national, regional, district, and sub-district/local participants	High
Kushitor et al. (78)	Community perceptions, involvement and how CHPS could be strengthened	Multiple, Rural	Qualitative, using focus group (20) with mothers and fathers of children under-5, adolescents without children and community leaders	High
Haykin et al. (79)	Perceptions of non-physician health workers on capacity to manage CVD at CHPS facilities	Upper East, Rural	Qualitative, using in-depth interview with 21 nurses and 10 nurse supervisors	High
Kweku et al. (80)	Challenges, capacity development priorities, and stakeholder perspectives on improving CHPS	Volta, Rural	Qualitative, using focus group (4) with health workers and community members	High
Kweku et al. (81)	Responsibilities, motivations, and challenges of CHPS community health management committees	Volta, Rural	Qualitative, using focus group (4) with CHVs	High
Wright et al. (82)	Community perceptions of gaps in CHPS maternal and child health services	Multiple, Rural	Qualitative, using focus group (53) with parents of children under-5, young men and women (15–24 years)	High
Bassoumah et al. (83)	Challenges to implementation and utilization CHPS	Northern, Rural	Qualitative exploratory, using in-depth interview (30) with CHOs, volunteers, and women receiving postnatal care	High
Sakeah et al. (84)	Selection procedures and roles of CHVs and CHMCs in CHPS	Upper East, Rural	Qualitative exploratory, using focus group (33) and in-depth interview (43) with health professionals and community members	High
**Mixed-methods studies, chronologically (*****n*** = **3)**				
Sacks et al. (85)	Domains of community health nurse satisfaction and motivation	Multiple, Rural	Cross-sectional, survey of 205 rostered CHNs, qualitative interviews (29) and focus groups (4) with selected CHNs	Medium
Yeboah and Francis (86)	Factors that facilitate or constrain community participation in CHPS	Central, Rural	Case study, using interview and informal discussion with community members, health volunteers, opinion leaders, CHOs, CHPS coordinator and Director of Health in municipality	Medium
Atinga et al. (87)	Community capacity to participate in CHPS implementation	Upper West, Rural and Urban	Exploratory sequential mixed-methods study, using in-depth interview (13), focus group (5) with key stakeholders of CHPS, and cross-sectional survey of 420 households	High

### 2.7. Data analysis

In accordance with our results-based convergent design, quantitative and qualitative findings were synthesized separately and then brought together in a final synthesis (29). For quantitative studies, effect sizes (Relative Risk, Odds Ratio, change in means), sample sizes and potential moderators (e.g., population characteristics) were summarized in tabular form. Due to the significant heterogeneity of studies, and with many studies drawing on the same longitudinal data set, we were unable to conduct the planned random-effects meta-analysis to estimate the effect size (and 95% confidence intervals) for each outcome. Instead, the key parameters reported in each study are presented in Tables 2–**4**.

**Table 2:** Reach, adoption, and implementation of CHPS by region^*^.

**Region**	**References**	**Population coverage and proportion of functional CHPS^#^ (year reported)**	**Utilization of CHPS**	**Trained CHOs at CHPS zones**	**Other staff and CHVs training**	**Proportion with CHMC (functioning)**
Ashanti	GHS (53)	CHPS zones with >5,000 population = 25.3% With basic equipment = 15.2% Functional CHPS = 7.8% (2018)	N/R	Zones with trained CHOs = 31.4%	Zones with trained CHVs = 75.3%	94%
Bono East	Wiru et al. (49)	12 Functional CHPS compounds sampled	12.3% said CHO absenteeism affected their use of CHPS	N/R	N/R	N/R
Brong Ahafo	GHS (53)	CHPS zones with >5,000 population = 22.8% With basic equipment = 30.7% Functional CHPS = 10% (2018)	N/R	Zones with trained CHOs = 35.4%	Zones with trained CHVs = 79.1%	97.4%
Central	Wood and Esena (43)	N/R	Of 175 respondents, CHPS utilized “Very often” by 2.9%, “Often” by 30.3%, “Not often” by 66.9%	N/R	N/R	N/R
	GHS (53)	CHPS zones with >5,000 population = 22.3% With basic equipment = 33.8% Functional CHPS = 11.1% (2018)	N/R	Zones with trained CHOs = 47.7%	Zones with trained CHVs = 77%	86.1%
Eastern	Naariyong et al. (41)	Within Brim North District: 11/49 areas were CHPS zones	N/R	N/R	N/R	N/R
	GHS (53)	CHPS zones with >5,000 population = 17.2% With basic equipment = 36.5% Functional CHPS = 6.5% (2018)	N/R	Zones with trained CHOs = 50.0%	Zones with trained CHVs = 82.8%	95.7%
	Amponsah et al. (55)	N/A: only areas with functional CHPS sampled	N/R	N/R	N/R	Three of 10 zones had regular VHM
Greater Accra	GHS (53)	CHPS zones with >5,000 population = 48.5% With basic equipment = 36.4% Functional CHPS = 4.7% (2018)	N/R	Zones with trained CHOs = 46.3%	Zones with trained CHVs = 33%	67.7%
Northern	Ferrer et al. (46)	N/R	11.8% (61/671)	N/R	N/R	N/R
	GHS (53)	CHPS zones with >5,000 population = 21.1% With basic equipment = 35.5% Functional CHPS = 10.8% (2018)	N/R	Zones with trained CHOs = 24.9%	Zones with trained CHVs = 93.7%	95.7%
Oti	Awoonor-Williams et al. (39)	By 2004, 30% of population exposed to CHPS	N/R	N/R	N/R	N/R
Upper East	Phillips (36)	By 2008, CHPS (combined) scaled up in all CHFP arms— <50% in cell1 (Zurugelu) areas, <60% in cell4 (comparison) areas, 100% in cell2 (nurse out-reach) and cell3 (combined) areas	N/R	N/R	N/R	N/R
	GHS (53)	CHPS zones with >5,000 = 9.1% With basic equipment = 47.8% Functional CHPS = 45.4% (2018)	N/R	Zones with trained CHOs = 54.3%	Zones with trained CHVs = 96%	97%
	Asuming et al. (38)	GEHIP increased coverage from 20 to 100% in intervention districts	N/R	100% in intervention districts	100% in intervention districts	N/R
Upper West	JICA (40)	36% of target number of functional CHPS zones by 2015, increasing from 24 in 2006 to 71 in 2009	N/R	N/R	160 CHOs trained	N/R
	Braimah et al. (52)	256 CHPS zones created as of 2017	N/R	N/R	N/R	N/R
	GHS (53)	CHPS zones with >5,000 = 3.3% With basic equipment = 55.2% Functional CHPS = 55.9% (2018)	N/R	Zones with trained CHOs = 83.2%	Zones with trained CHVs = 97.5%	93.6%
Volta	Ferrer et al. (46)	N/R	31.3% (228/685)	N/R	N/R	N/R
	GHS (53)	CHPS zones with >5,000 = 17.9% With basic equipment = 18.4% Functional CHPS = 6.7% (2018)	N/R	Zones with trained CHOs = 39.2%	Zones with trained CHVs = 73.9%	79.3%
	Kweku et al. (56, 57)	Central Tongu 15/18 demarcated CHPS zones were functional Nkwanta South 21/25 demarcated CHPS zones were functional	Central Tongu 53.8% Nkwanta South 76.6% Both districts 65.2%	N/R	N/R	N/R
Western	GHS (53)	CHPS zones with >5,000 population = 21.0% With basic equipment = 39.1% Functional CHPS = 13.2% (2018)	N/R	Zones with trained CHOs = 45.1%	Zones with trained CHVs = 72.4%	89.2%
	Maly et al. (54)	Only CHPS zones (24) with physical structure were sampled	N/R	Mean 3 CHOs per CHPS zone (range 1–8)	N/R	22/24
National	Johnson et al. (44)	2009–2011 CHPS zones doubled from 868 to 1,675 (functionality not specified)	N/R	N/R	N/R	N/R
	GHS (53)	CHPS zones with >5,000 = 21.9% (national average CHPS zone population = 3,821) Of the 5,918 CHPS zones surveyed, 13% were considered functional, 31.4% had basic equipment	N/R	Zones with trained CHOs = 42.4%	Zones with trained CHVs = 76.2%	89.8%

^*^Bono, North East, Savannah, Western Northern–no quantitative results relating to adoption or implementation of CHPS from these regions [GHS (53) validation survey report presents results for national-level and by region, but not according to the new list of regions].

^#^Functional CHPS means all steps completed except construction of compound, motorbike training, procure bicycle, procure drug kits and volunteer supplies.

**Figure 3:**
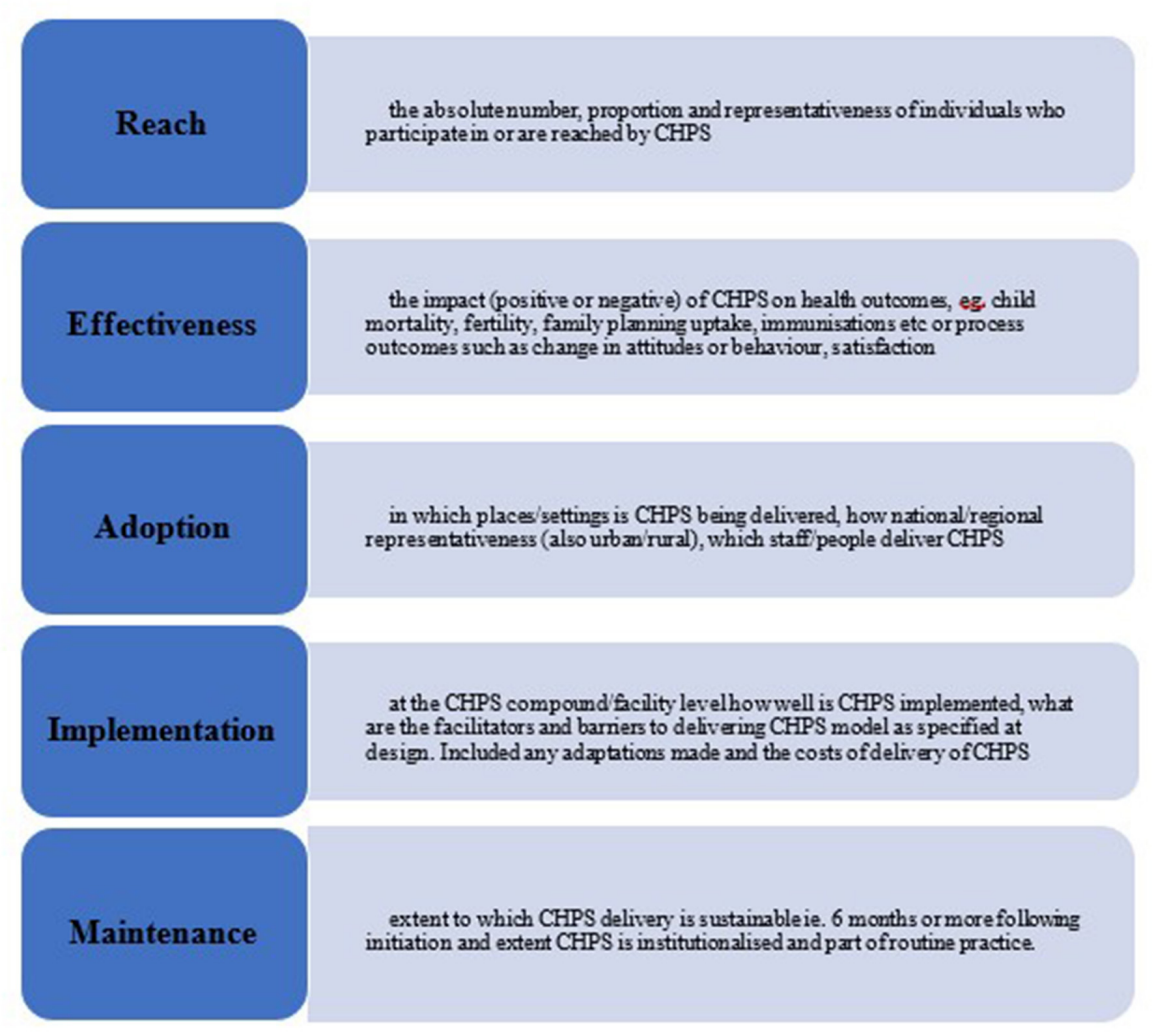
RE-AIM categorizations used in the review of CHPS studies.

Data from qualitative studies were extracted and analyzed using the RE-AIM framework. The RE-AIM framework has been used extensively (88) to evaluate public health interventions and aims to understand not only effectiveness (E and our objective 1), but also who is reached (R) by the intervention, how far it has been adopted (A) in different settings and by different health workers (addressing our objective 2), and lessons on implementation (I) and maintenance (M) which refers to the sustainability of the programme (addressing our objective 3, see Figure 3). Segments (commonly sentences) within the qualitative findings were coded against the five RE-AIM domains independently by two reviewers and arbitrated by a third reviewer. Once all findings had been coded, the segments from each study were combined and reorganized under the RE-AIM domains. Segments were then compared and where one segment was clearly articulating the same issue as a segment from another study, these were grouped together and assigned a heading that represented all grouped and single segments. These were color-coded to illustrate issues that occurred frequently and less frequently in the synthesized findings. Issues occurring less frequently should not be seen as less important, merely that they were identified less frequently in published studies (see **Figures 5**, **6**).

Qualitative and quantitative findings from mixed methods studies were included in the respective qualitative and quantitative synthesis. Any meta-inference from mixed methods studies was included in the qualitative synthesis. The final synthesis of quantitative and qualitative data was conducted according to the RE-AIM framework. We identified and confirmed any key lessons, commonalities, and any contradictions by returning where necessary to included studies and quality assessments.

## 3. Results

### 3.1. Study selection and characteristics

**Figure 4:**
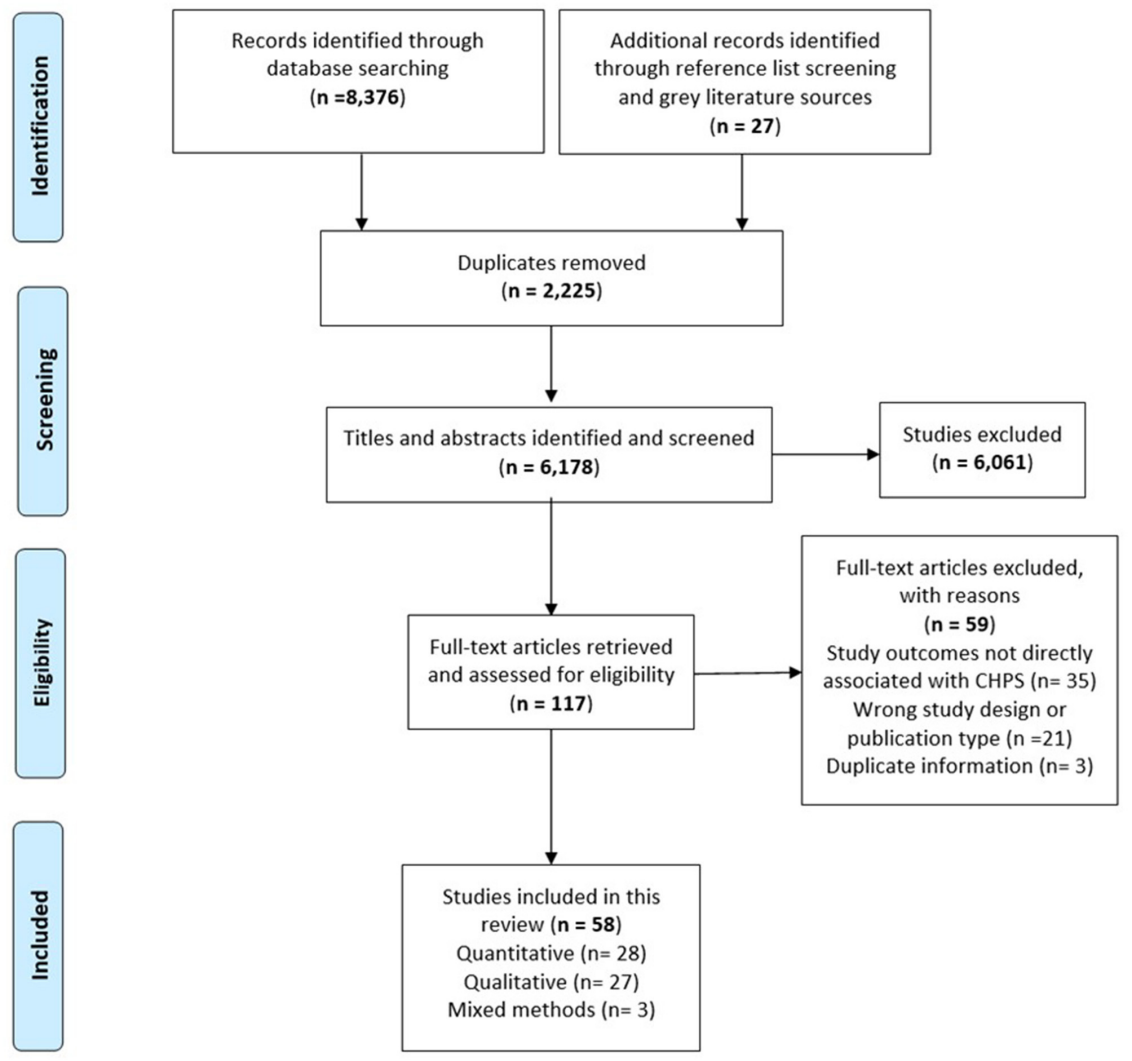
PRISMA flow diagram of the CHPS review.

A total of 8,376 records were initially identified through the electronic searches with an additional 27 papers identified through reference list screening and gray literature sources, of which 2,225 were duplicates and removed. Following screening, 117 full text papers were assessed for eligibility, with 59 excluded with reasons, leaving 58 papers included in the final synthesis and analysis (see the PRISMA flow chart in Figure 4). The final synthesis included 58 studies, 28 of which were qualitative, 27 quantitative, and three mixed methods studies. Details of the quantitative findings are presented in the following tables: Table 2 presents a summary of the quantitative results relating to the domains of Reach, Adoption and Implementation; Table 3 presents quantitative results of effectiveness in improving child mortality and fertility; and Table 4 presents effectiveness of other outcomes measured in the included studies on family planning, maternal and child health. Qualitative findings are integrated with key quantitative results under the RE-AIM domains in the text below.

**Table 3:** Studies of CHPS assessing effectiveness in improving child mortality and fertility.

**References**	**Context Study design Sample**	**Intervention**	**Fertility, parity progression and contraceptive prevalence**	**Under-5 child mortality rate (0–59 months)**	**Neonatal mortality rate (first 1 month of life)**	**Infant mortality rate (0–11 months)**	**Early child mortality rate (0–23 months)**	**Late child mortality rate (24–59 months)**
Debpuur et al. (26)	Kassena-Nankana District Pilot and 4-arm plausibility trial phases (baseline 1993 and plausibility trial 1996–1999) *N* = 8,998 women (15–49 years)	Arm 1: Volunteers and community engagement Arm 2: CHO located in sub district health center <10 km from rural households Arm 3: Both volunteers and CHOs (i.e., CHPS) Arm 4: Neither/Comparison Analysis of Navrongo Demographic Surveillance System (NDSS) data to assess impact on family planning knowledge, use and fertility	Contraceptive prevalence rises from 3.4% in 1993 to 1999: Arm 1 (Vol) = 6.0%; Arm 2 (CHO) = 6.0%; Arm 3 (Vol + CHO, CHPS) = 8.2%; Arm 4 (Comparison) = 6.0% Total fertility rate dropped in all 4 arms OR for parity progression compared to Arm 4 (Comparison) from 1993 to 1999: Arm 1 (Vol) = 0.81, *p* < 0.05; Arm 2 (CHO) = 0.85, *p* < 0.05; Arm 3 (Vol + CHO, CHPS) = 0.77, *p* < 0.05	NA	NA	NA	NA	NA
Phillips et al. (34)	Kassena-Nankana District Plausibility trial with four arms and 9 time points between 1996 and 2003 *N* = 139,000 individuals	Same arms as above	Fertility rate reduced by 15.0% in Arm 3 (Vol + CHO, CHPS) compared to Arm 4 (Comparison).	Arm 3 (Vol + CHO, CHPS) = 224–100 deaths per 1,000 person-years; Arm 4 (Comparison) = 212 to 145 deaths per 1,000 person-years No significant difference between Arm 1 (Vol) or Arm 2 (CHO) and Arm 4 (Comparison); 95% CI or *p*-value not presented	NA	NA	NA	NA
Pence et al. (24)	Kassena-Nankana District Plausibility trial (1 July 1993−30 April 2000) *N* = 52,801 children and 52,801 mothers	Same arms as above	NA	(0–59 months) Significant positive effect: Arm 2 (CH0) Rate Ratio = 0.86 (95% CI = 0.74, 0.99) No significant difference in before/after analysis: Arm 1 (Vol), Arm 3 (Vol + CHO, CHPS) and Arm 4 (Comparison)	NA	(0–11 months) No significant difference in before/after analysis in any arm. But greater declines seen in Arm 2 (CHO) and Arm 3 (Vol + CHO, CHPS): Arm 1 (Vol) = −11%; Arm 2 (CHO) = −43%; Arm 3 (Vol + CHO, CHPS) = −33%; Arm 4 (Comparison) = −13%	(12–23 months) Significant negative effect: Arm 1 (Vol) Rate Ratio = 2.35 (95% CI = 1.52, 3.63) No significant difference in before/after analysis: Arm 2 (CHO), Arm 3 (Vol + CHO, CHPS) and Arm 4 (Comparison).	(24–59 months) Significant positive effect: Arm 2 (CH0) Rate Ratio = 0.61 (95% CI = 0.42, 0.88)
Phillips (36)	Kassena-Nankana District Plausibility trial, assessed the impact period (1995–2001) and CHPS scale-up period (2004–2010) *N* = 47,036 women (15–49 years)	Same arms as above; further arms added in scale up: Arm 5 (Comparison for scale-up): Volunteer services added to Arm 4 Arm 6: CHOs added to Arm 4 Arm 7: Volunteers added to Arm 2 (CHO only) Arm 8: CHOs added to Arm 1 (Vol only)	Total fertility rate in impact period (1995–2001): Arm 1 (Vol) = 5.01–4.40; Arm 2 (CHO) = 5.75–5.34; Arm 3 (Vol + CHO, CHPS) = 4.94–4.33 Arm 4 (Comparison) = 5.06–4.89 Significant difference between Arm 3 (Vol + CHO, CHPS) and Arm 4 (Comparison) in 2001: Linearized hazard ratio = 0.85 (95% CI = 0.79, 0.92); non-significant in other arms. In scale-up period (2004–2010): Arm 1 (Vol) = 4.24–3.59; Arm 2 (CHO) = 4.94–4.72; Arm 3 (Vol + CHO, CHPS) = 4.03–3.71; Arm 4 (Comparison) = 4.69–4.07 By 2010, significant difference between Arm 4 (Comparison) and Arm 1 (Vol) = 0.88 (0.81, 0.96); and New Arm 7 (Volunteers added to CHOs) = 1.11 (1.02, 1.21)	NA	NA	NA	NA	NA
Bawah et al. (37)	Kassena-Nankana District Plausibility trial (January 1, 1995 to December 2010) *N* = 94,599 under 5 children	As above four arms, analysis of Navrongo Demographic Surveillance System (NDSS) data to identify relationship between wealth/education and child mortality in the 4 arms. Age-conditional proportional hazard analysis	NA	All arms showed improvements, but only Arm 3 (Vol + CHO, CHPS) significantly improved mortality among the poorest and least educated, over all time periods: HR by 2008–2010 Arm 1 (Vol) HR = 0.98, NS; Arm 2 (CHO) HR = 1.11, NS; Arm 3 (Vol + CHO, CHPS) HR = 0.67, *p* < 0.01; Arm 4 (Comparison) HR = 1.00	NA	NA	NA	NA
Awoonor-Williams et al. (39)	Nkwanta District 2002 district level survey *N* = 831 women (15–49 years)	Cross-sectional survey of CHPS and non-CHPS zones, using logistic regression models to assess the effect of CHPS exposure on health indicators	Adjusted risk ratio for CHPS generating knowledge of modern contraception = 1.82, *p* < 0.01 and for use of modern contraceptives among those who reported knowledge = 3.33, *p* < 0.01	NA	NA	NA	NA	NA
Bawah et al. (37)	Upper East Region GEHIP (A 5-year trial launched in 2010, to test means of accelerating CHPS) *N* = 7,044 under-5 children and 5,914 women	Clusters: four treatment and seven contiguous comparison districts	NA	It is not possible to obtain an overall estimate of mortality for all children under 5 because the mortality hazard ratio varies by age	GEHIP reduced neonatal mortality by approximately one half (HR = 0.52, 95% CI = 0.28, 0.98, *p* = 0.045).	No significant difference between GEHIP and control (HR = 0.72; 95% CI = 0.30, 1.79; *p* = 0.480)	NA	NA
Asuming et al. (38)	Upper East Region GEHIP (A 5-year trial launched in 2010, to test means of accelerating CHPS) *N* = 5,914 women (15–49 years)	Clusters: four treatment and seven contiguous comparison districts	Contraceptive prevalence rises by 64.40% in intervention and 7.60% in comparison districts between baseline and end line (2011–2015) aOR for use of modern contraceptives among currently married women in intervention vs. comparison district = 1.79 (95% CI = 1.32, 2.44), *p* < 0.01	NA	NA	NA	NA	NA

**Table 4:** Other outcomes: family planning, maternal, and child health.

**References Context Study design Sample**	**Intervention**	**ANC**	**Delivery attended by a medical professional or skilled birth attendant**	**PNC**	**Health knowledge (including knowledge of contraception)**	**Contraception indicators**
Debpuur et al. (26) Kassena-Nankana District 4-arm plausibility trial *N* = 8,998 women (15–49 years)	Arm 1: Volunteers and community engagement Arm 2: CHO in health center <10 km from households Arm 3: Both (CHPS) Arm 4: Neither/Comparison Analysis of NDSS data to assess impact	NA	NA	NA	OR for modern contraception knowledge compared to Arm 4 (Comparison) from 1993 to 1999: Arm 1 (Vol) = 0.72, *p* < 0.05; Arm 2 (CHO) = 0.94, NS; Arm 3 (CHPS) = 1.28, NS	OR for identifying source for contraception compared to Arm 4 (Comparison) from 1993 to 1999: Arm 1 (Vol) = 0.67, *p* < 0.05; Arm 2 (CHO) = 0.60, *p* < 0.01; Arm 3 (CHPS) = 1.19, NS
Awoonor-Williams et al. (39) Nkwanta District 2002 district-level survey *N* = 831 women (15–49 years)	Cross-sectional survey of CHPS and non-CHPS zones, using logistic regression models to assess effect of CHPS exposure on health indicators	Adjusted OR for CHPS exposure and ANC attended by health professional = 1.79, *p* < 0.05	Adjusted OR for CHPS exposure vs. non-exposure = 1.79, *p* < 0.05	Adjusted OR for CHPS exposure and PNC attended by health professional = 3.20, *p* < 0.01	Adjusted OR for CHPS exposure and unprompted knowledge of one or more family planning methods = 2.12, *p* < 0.01	NA
Naariyong et al. (41) Birim North District 2010 survey *N* = 600 mothers (15–49 years)	Cross-sectional survey of CHPS and non-CHPS zones, using logistic regression models to assess effect of CHPS exposure on health indicators	Adjusted OR for CHPS exposure with: Full utilization of ANC services = 2.73 (95% CI 1.68–4.43), *p* < 0.001 Receipt of malaria Prophylaxis = 3.73 (95% CI 1.73–8.04), *p* < 0.05 Tested for HIV Infection = 4.49 (95% CI 2.37–8.51), *p* < 0.001	NA	NA	Adjusted OR for CHPS exposure and index of knowledge about pregnancy danger signs = 1.17 (95% CI 0.69–2.00), NS	NA
Johnson et al. (44) National 2003 and 2008 Ghana Demographic and Health Survey (GDHS) *N* = 4,349 births	Secondary analysis of GDHS data with logistic regression Models to examine the effect of proximity to health facilities and CHPS on use of skilled care at birth	NA	Adjusted OR for uptake of skilled birth care with CHPS-only = 1.40 (95% CI 0.61–3.24), NS For CHPS and health facility within 8 km = 1.56 (95% CI 1.04–2.36), *p* < 0.05	NA	NA	NA
Ferrer et al. (46) Volta and Northern Regions 2014 household survey *N* = 1,356 carers of children under-5	Survey conducted two and eight years after iCCM in Volta and Northern Regions respectively, and more than 10 years of CHPS in both regions	NA	NA	NA	Volta: Adjusted OR for carers to identify at least two signs of severe diarrhea after messages from CHPS = 3.6 (95% CI 1.4–9.0), *p* 0.02 Northern: receiving messages from CHPS was not associated with knowledge	NA
Sakeah et al. (50) Builsa and West Mamprusi Districts 2016 household survey *N* = 650 women who had delivered in the past 5 years	Survey conducted at CHPS zones in both districts	87% of the women reported having had at least four ANC attendance (Bulisa = 93.1%, West Mamprusi = 80.8%)	66.3% were supervised by a skilled attendant during child birth (Bulisa = 75.4%, West Mamprusi = 57.2%)	62.3% had attended PNC at least three times (Bulisa = 90.1%, West Mamprusi = 34.5%)	NA	NA
Asuming et al. (38) Upper East Region GEHIP (A 5-year trial launched in 2010, to test means of accelerating CHPS) *N* = 5,914 women (15–49 years)	Clusters: four treatment and seven contiguous comparison districts	NA	NA	NA	NA	Crude OR for unmet need for modern contraceptives among currently married women in intervention vs. comparison district = 0.85 (95% CI 0.64–1.12)

### 3.2. Study settings

**Figure 5:**
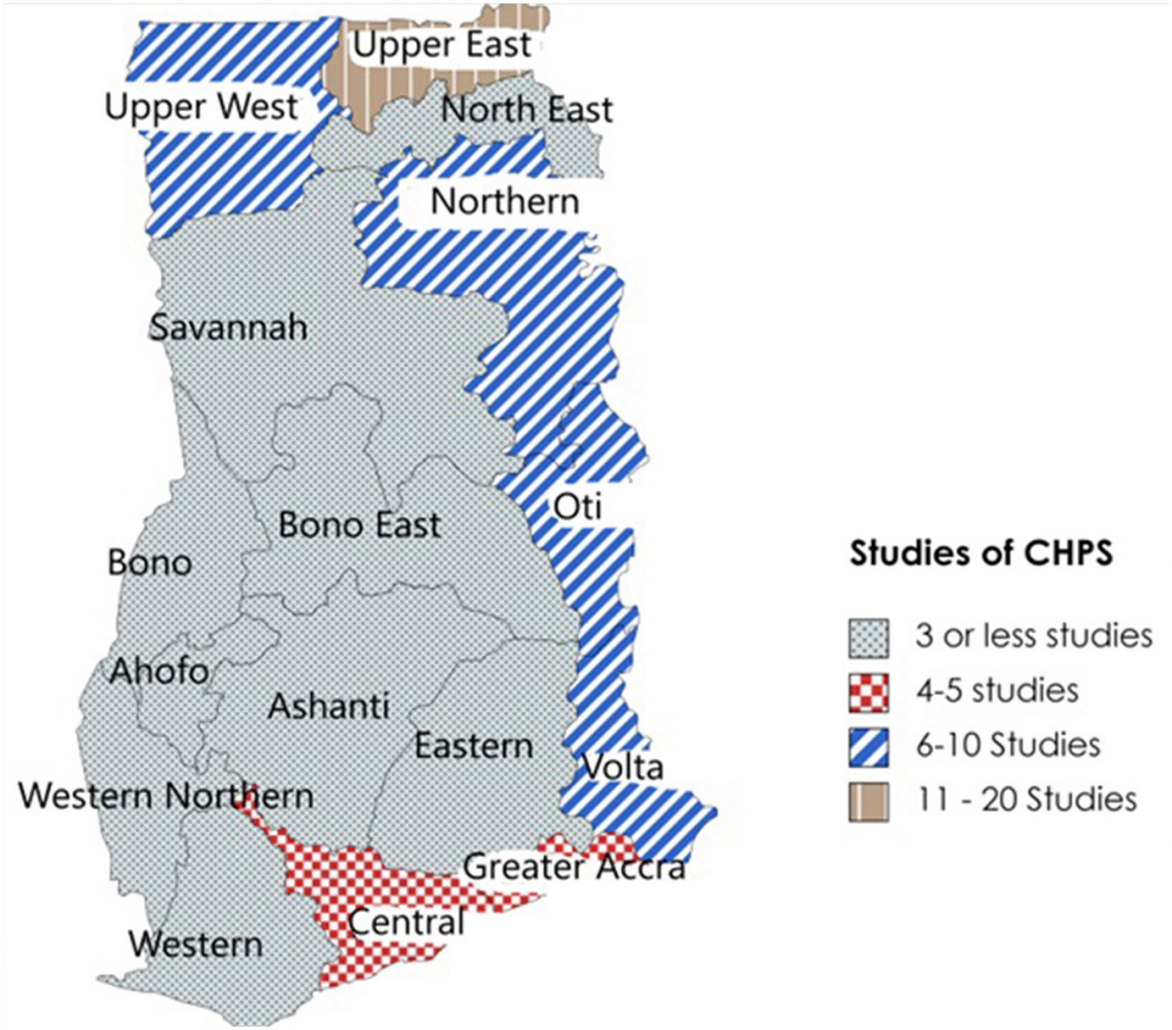
Regional distribution of CHPS studies included in the review.

The geographical spread highlights the uneven distribution of studies assessing CHPS, with the majority conducted in the Upper East Region, where the original Navrongo Experiment was located (see Figure 5). While most studies focused on CHPS in rural settings, some papers have assessed CHPS implementation in urban areas, including three qualitative studies (51, 61, 73), and one quantitative study conducted only in urban areas (55).

### 3.3. Adoption of CHPS within different settings

#### 3.3.1. Low functionality in remote rural and urban areas

Guided by the RE-AIM framework, “adoption” refers to the places and settings in which the CHPS programme is being delivered and thus highlights geographical regions or types of areas where adoption has been limited. Following the launch of the national policy to scale up CHPS in 1999, there has been a focus in the literature on increasing the coverage of the programme (see Table 2). National level studies identified that between 2009 and 2011, functional CHPS compounds doubled from 868 to 1675 (44).

A process of declassification of “non-functional” CHPS zones took place throughout the country in 2018. CHPS zones were classed as non-functional when CHPS compounds were found to be non-existent or essential staff and equipment were not available (53). This was found to be particularly apparent in remote rural areas, with the North East and Northern regions having only 22. Four percent and 33.8% of CHPS zones functioning effectively (53). Adoption was also challenging in urban areas, for example in the Greater Accra region only 672 of the 834 zones were termed “functional,” and only 539 of them had basic equipment to provide services (53). As a result of this declassification, the GHS reported that by September 2019, there were 5,155 functional zones, 2,467 zones with compounds, and 3,160 with basic equipment nationally (53).

#### 3.3.2. Resources and leadership required for adoption

**Figure 6:**
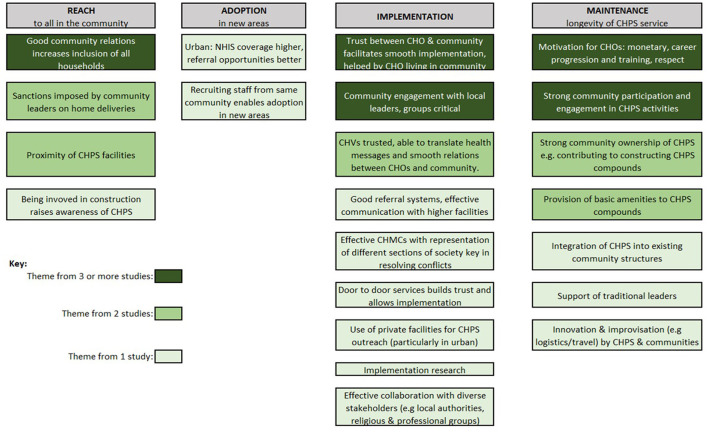
Facilitators from qualitative studies.

**Figure 7:**
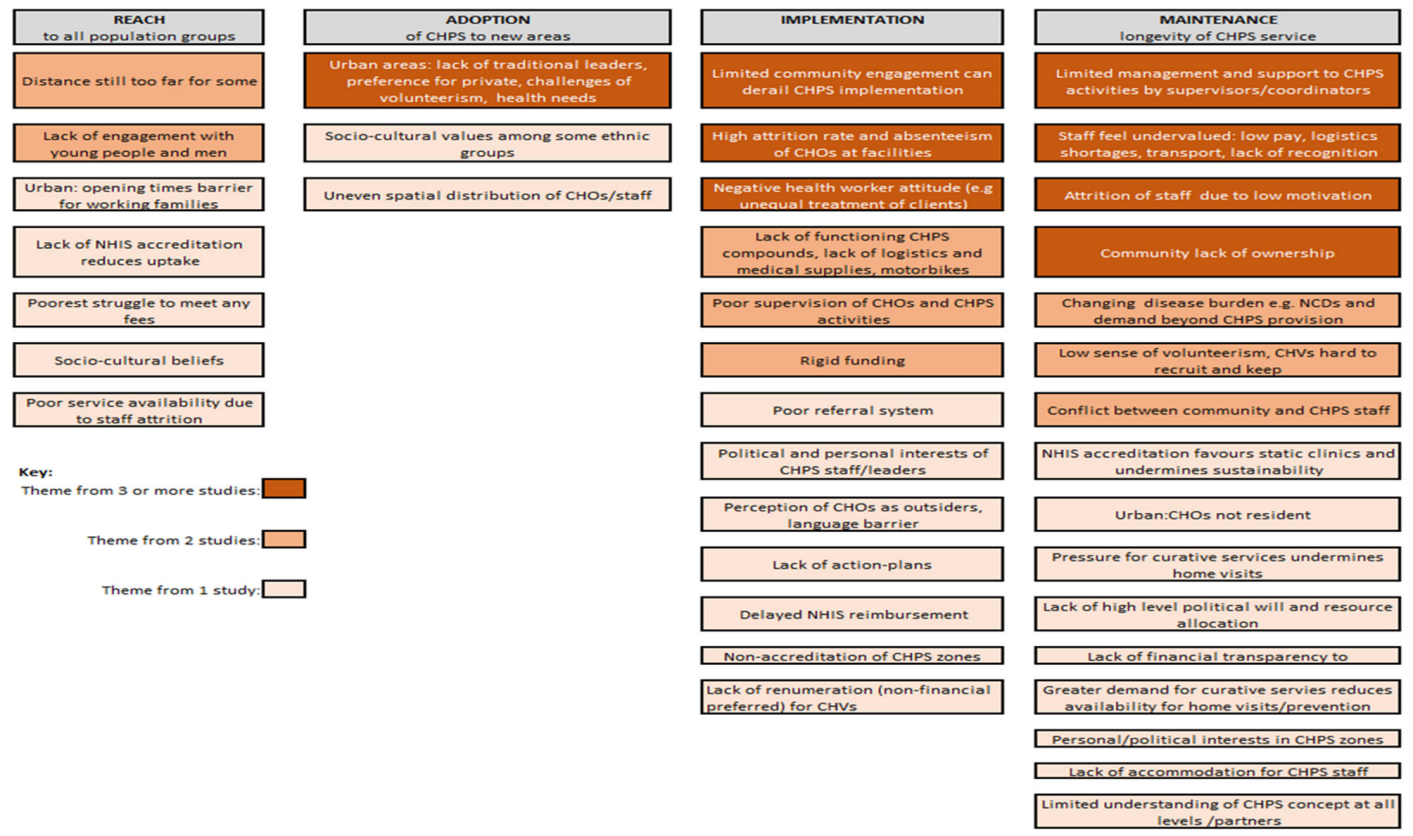
Barriers from qualitative studies.

Qualitative studies highlighted the facilitators and barriers to adoption of CHPS within different geographical settings (see Figures 6, 7). For under-served rural areas there were particular challenges due to the uneven distribution of CHOs (67) and inadequate accommodation for CHOs (67, 72, 85), while recruitment of staff from the communities they serve aided adoption of CHPS in these areas (63). The majority of qualitative studies cited limited investment in the development of new CHPS compounds with insufficient supplies, equipment and infrastructure to deliver CHPS services as a major barrier to wide scale adoption. Authors explained this was due to a lack of financial resources within Ghana's health sector (58, 67) which impeded actions to scale up CHPS from sub-district to national level (65). Nyonator et al. (6) found that with some creative mobilization of resources, and particularly with political support, including politicians contributing funds to CHPS, districts were able to establish functioning CHPS zones (58, 63). However, when there was a low level of awareness of the principles of CHPS (including shared ownership between government and communities) (59), and a strong political motivation for building CHPS compounds during local elections without ensuring they were equipped and staffed (67), the zones were not able to function.

#### 3.3.3. Socio-economic structures in urban areas challenge adoption of the rural model

Despite the potential strengths of the urban setting, such as better roads and facilities suitable for referral of emergency cases (51, 74) challenges specific to the adoption of the CHPS model in urban areas were found. These included the lack of traditional leadership structures, lack of trust and limited home-visiting and engagement (51). These challenges were exacerbated by the fact that staff often did not come from or live in the communities in which they work (73), due to the difficulty in finding accommodation in the area (51, 61). The need to pay volunteers due to the opportunity cost they face (61), declining shared community values, particularly among socially alienated young people (87), and a preference for private facilities were also reported challenges. In addition, a changing disease burden with increases in non-communicable diseases and subsequent shifting demand for services by urban residents raised further challenges to the adoption of the original model (61), particularly given CHOs do their field training only in rural CHPS zones (73). Nevertheless, attempts to adapt the model to link in with private facilities which could then become CHPS outreach points for urban communities was identified as a potential facilitator to the adoption of the CHPS model within urban areas (51).

### 3.4. Reach of CHPS

#### 3.4.1. Variation in reach

Within the RE-AIM framework, “reach” focuses on the absolute number, proportion and representativeness of individuals who participate in or are reached by CHPS. Given the aim of CHPS to increase access for all to health care, many of the quantitative studies assessing CHPS have looked at overall coverage (see Table 2) or utilization across the population through cross-sectional household surveys. Findings varied across regions with rates of utilization of 76.7% in Nkwanta South Municipal (Oti Region) and 53.8% in Central Tongu District (Volta Region) (56), whereas Wood and Esena's earlier study in Central Region found lower rates with 66.9% reporting rare use of CHPS (43). Ferrer found 11.8% in Volta region and 31% of the population in Northern region utilizing CHPS for childhood illnesses (46). Johnson's national analysis using 2003 and 2008 Demographic and Health Survey data found only 9.9% of all births were in communities within 8 km of CHPS (44). Given the different methods, tools, and target populations of these studies, results are not comparable, but do indicate the variability of reach of the CHPS programme across Ghana.

#### 3.4.2. Inequities in reach

Studies identifying *who* in the population CHPS reaches were more limited. While quantitative studies have explored whether there is a social gradient in health improvements in CHPS areas (37), few studies quantified whether particular groups within communities were more or less likely to be “reached” by the programme. In the Upper East Region, ethnic and educational differences were found to undermine equal reach, with women of the Nankana ethnic group significantly disadvantaged in accessing CHPS for delivery compared to those within Kassena communities, possibly due to the former's more traditional beliefs about childbirth (66). Differences in reach to specific ethnic and religious groups were also found in Nkwanta, with Christian and Muslim women more likely to receive safe-motherhood care than women who identified as traditionalists or with no religion (39).

#### 3.4.3. Reaching young people and men

Further insights on “reach” from the qualitative studies include the observation that young people (78, 87) were frequently overlooked by the CHPS programme. There were mixed findings on the ability of CHPS to reach fathers with several studies identifying Father-to-Father Support Groups as a valuable mechanism for increasing male knowledge on health issues (72), and male involvement being evident in family planning activities of CHPS (45, 89). Others found the CHPS programme rarely reached men with many seeing the programme as a “women's thing” (78) and traditional gender norms around pregnancy and childbirth influencing the nature and level of male involvement in maternal health and CHPS more broadly (62), and this was highlighted in family planning programmes in Southern Ghana (90). Reaching particular groups of vulnerable individuals far from the CHPS compound was a common challenge described in a number of qualitative studies, especially in relation to people with cardio-vascular disease (79), and maternal health care where women challenged the accepted notion that 5 km should be considered walking distance when seeking maternal services without access to good roads and any means of transport (69).

### 3.5. Effectiveness of CHPS

#### 3.5.1. Mortality and family planning

Since the inception of CHPS, effectiveness studies have focused on child mortality and fertility as primary health outcomes. Many studies have also assessed key “process outcomes” such as uptake of antenatal care visits and institutional deliveries, immunizations and child health programmes (44, 46, 47, 55). Studies with a low risk of bias reporting the effectiveness of the CHPS programme in health outcomes are shown in Table 3. These studies all use data from the Navrongo Demographic Surveillance System (NDSS) 1990–2010 and compare four interventions implemented in Kassena-Nankana district, Upper East Region: (1) Volunteers (Zurugelu), (2) Nurse only, (3) Nurse + Volunteers, and (4) “unexposed” areas. Three studies assessed under-5 mortality (24, 34, 37). The most detailed analysis, which analyzed mortality over time and identified interactions with wealth and education found under-5 mortality improved over time in all areas, but Volunteers alone and CHO alone benefitted the better off and educated. Only the combination of CHO and volunteers significantly reduced under-5 mortality in the poorest and least educated (37).

One study (24) assessed infant mortality but found no significant difference between the four interventions from baseline, but greater declines were seen in CHO (243%) and CHO plus Volunteer areas (233%) than in the volunteer only (211%) and comparison areas (213%).

Three studies used the NDSS data and four-arm trial design to assess outcomes of family planning including change in fertility rate (26, 34, 36). Given the context of Kassena-Nankana district where the “fertility transition” had not begun in early 1990's (i.e., 3.4% in 1993), a rise in contraceptive use and drop in fertility rate was found in all four intervention areas, but the odds of parity progression reducing from 1993 to 1999 were highest in the CHO plus Volunteer arm (see Table 3).

#### 3.5.2. Maternal health

The results of studies reporting outcomes associated with improved health are shown in Table 4. In Nkwanta district, the presence of a CHPS zone was identified as increasing the odds for delivery attended by medical professional [OR1 = 1.74 (*p* < 0.01), OR2 = 1.79 (*p* < 0.05)] and for postnatal care from a medical professional [OR1 = 3.09 (*p* < 0.01), OR2 = 3.20 (*p* < 0.01)] (39). Assessment of national DHS data found that the presence of a CHPS zone in addition to a health facility resulted in increased odds of care by a skilled birth attendant by 56% (44). In Brim North, Eastern Region, CHPS exposure was found to be positively associated with receipt of ANC (OR 2.73 (95% CI 1.68–4.43) compared to participants in non-CHPS areas and these improvements in the provision of four ANC visits (75.4% in CHPS compared to 72.3% in non-CHPS) from a trained provider (96.3% in CHPS and 90.3% in non-CHPS) increased the odds of receiving an HIV test and anti-malarial prophylaxis (41).

#### 3.5.3. Child health

In terms of child health programmes, CHPS has been compared with integrated community case management (iCCM) in the Volta and Northern regions of Ghana. Differences in effectiveness between the two interventions were found in each region with health messaging from CHPS found to be associated with identification of severe diarrhea by parents in Volta and prompt care seeking in Northern Region (47). Cost-effectiveness analysis found that appropriate diagnosis and treatment of malaria, diarrhea and pneumonia were more cost-effective under iCCM than CHPS in the Volta Region (48).

#### 3.5.4. Accessibility and acceptance

Qualitative studies frequently highlight positive perceptions of effectiveness of CHPS at community level, with respondents acknowledging the programme's significant role in making basic health services more accessible for women and children, allowing them to benefit from immunization, ante- and postnatal care, health education, family planning, referral of severe disease conditions and school health visits, in addition to improving health outcomes in their respective zones (61, 73, 82).

Participants in several qualitative studies also highlighted the critical role CHPS has played in changing negative perceptions of some health services, particularly family planning, through improved knowledge of the side effects of contraception (45, 60, 61). This increased acceptance of family planning was identified as creating a shift in perceptions of the ideal family size, with spacing births seen as desirable, although some women still reported keeping their use of contraceptive secret from their husbands (70).

### 3.6. Implementation of CHPS: barriers and facilitators

#### 3.6.1. Trust and engagement

Both quantitative and qualitative studies identified barriers and facilitators to the implementation of the CHPS model as specified at design. Two inter-related themes that consistently emerged across studies and settings was the need for trust between CHPS staff and communities for smooth implementation, and vital to this was strong community engagement (see Figure 6). When CHOs lived within the communities they service, these good relationships could develop (66, 71, 77). Volunteers played a vital bridging role between CHOs and communities, often facilitating implementation with their diplomacy skills, as well as offering practical support by running errands for CHOs and sometimes taking CHOs for home visits on their motorbikes (60).

Community engagement organized through local leaders and women's groups to solicit their support for CHPS was frequently identified as critical for effective implementation in the rural studies (51, 57, 58, 63, 64, 68, 77, 82, 87). Where the engagement component of CHPS were adapted sensitively to the local context, implementation was more successful. For instance, in Nkwanta, which has a more complex ethnic composition than the original Navrongo communities, the engagement process was adapted so instead of relying on traditional leaders to organize community action in CHPS as had been done in the Navrongo model, leaders were rather identified among elected officials, teachers and clerics (45). A strong CHMC with membership able to resolve any conflicts between health staff and community members has also been identified as important for CHPS implementation in such rural settings (61). One study that quantified community engagement within the CHPSplus (CHPS+) intervention in Volta region found that 48.9% of the 1,000 respondents were actively involved, including through the identification of resources, organizing durbars and preparing sites for outreach services, and that involvement in these activities was associated with positive perceptions of CHPS (80).

There was much consistency in the barriers to implementation identified in the qualitative studies (see Figure 7) and the majority cited limited community engagement as a key underlying cause of poor CHPS implementation (58, 62, 65, 77). Lack of engagement specifically led to CHMCs that were not sufficiently active to provide the support and problem-solving needed for implementation (53). Several studies identified low volunteer motivation, particularly in urban areas, where communities were not sufficiently engaged (73).

#### 3.6.2. Organizational collaboration

Beyond the community level, effective implementation was characterized by careful collaboration with diverse stakeholders but particularly local authorities, religious organizations and professional groups and associations. This helped to facilitate ongoing operations such as establishing referral systems to higher facilities, which promotes the use of CHPS services (66). The importance of outreach services, particularly door to door services has been identified by several studies as key for both delivering services (60, 61), and also in building trust (56, 77).

#### 3.6.3. Accommodation and logistics

From the health systems perspective, the most frequently reported barriers to implementation were the lack of provision of accommodation for CHOs, logistics and facilities to ensure a functioning CHPS zone and this was found both in rural and urban areas (51, 57, 65, 71, 73, 78, 82, 87). Lack of accommodation for the CHOS within the community was a particular challenge undermining both service delivery and the level of trust between CHOs and community members (66, 77, 82). Within urban areas, where land is scarce, this was a particular challenge with CHOs having to commute into their areas of work (61, 73). In rural areas, the recruitment of CHOs from outside the communities and who may not therefore share a common language was identified as undermining implementation both by CHOs and by communities (85). The wider implications of limited resources were evident, with the lack of motorbikes and provision of funds for their running and maintenance undermining CHOs' ability to undertake home visits leading to more clinic-based static services and reduced trust and engagement with households (59). Frequent stock-outs of essential medicines including contraceptives was noted by CHOs and women in the communities as a challenge that undermined reliable service delivery (43, 70) with shortages of medicines reported by 41.5% of survey respondents in Bono East Region (49).

#### 3.6.4. Supervision, training, and referrals

Further health systems challenges were noted, particularly the limited supervision from CHPS coordinators at sub-district level and from higher levels (73). Cited reasons for this in both rural and urban areas were the lack of available transport and human resources (53, 65, 73). Referral systems were frequently found to be lacking (57) and CHOs expressed a wish for further training (85) not only in clinical skills such as midwifery (59) and childhood illnesses (47), but also to improve support to volunteers, planning and data collection (74). The limitations to facilities, accommodation, resources, support and training were frequently cited as a cause of the low motivation, with just over 50% of CHOs stating they were satisfied with their role (85). Low levels of motivation and negative attitudes among CHOs were identified as a cause of favoritism and unequal treatment of clients, and affected the effective implementation of CHPS (56, 77, 78). Subsequently, a high attrition rate of CHOs was identified in several of the qualitative studies (57, 72–74).

### 3.7. Maintenance of CHPS

#### 3.7.1. Planning, budgets, and insurance

The RE-AIM framework defines “maintenance” as the extent to which CHPS can be delivered sustainably for at least 6 months or more following initiation. This domain allows exploration of the extent to which CHPS has become institutionalized and part of routine practice. The included studies identified several issues that undermined the sustainability of CHPS services over time. Low motivation and high absenteeism of CHOs, changing disease burden, increasing demands and expectations of communities beyond the prescribed service package of CHPS, linked with the implementation issues identified above have all been identified as a threat to sustainability of CHPS (67, 71, 72, 79, 82, 85). The non-accreditation of elements of the CHPS programme, particularly home-visits under the National Health Insurance Scheme (NHIS) has also been identified as distorting delivery to favor clinic-based services, therefore undermining the outreach and community engagement components of CHPS in the long term (34, 60, 77, 82). Even where NHIS accreditation does exist, the delayed NHIS reimbursement undermines continued delivery of service (53). The changing disease burden has also been identified as a threat to sustainability of CHPS (79, 82) and particularly the increasing demands and expectations of communities beyond the prescribed service package of CHPS (67).

However, more fundamental organizational issues were also highlighted as barriers to CHPS maintenance, including a lack of action planning, and more crucially limited budget, with the Ministry of Health and GHS having no specific budgets to support the CHPS programme (58), reportedly linked to a lack of high-level political will and resource allocation specifically to CHPS (49).

#### 3.7.2. Community collaboration and ownership

Conversely, in areas where CHPS has managed to engage communities, particularly with strong support from traditional leaders (56), integration within existing community structures that predated the establishment of CHPS in the community (64), and initial community contributions to constructing CHPS compounds (56, 68), CHPS programmes seemed able to flourish and sustain activities. Similarly, where CHOs reported feeling motivated and respected by communities and supervisors (66, 85), with basic amenities provided in CHPS compounds (57, 66) and adequately trained (53), CHPS services were maintained.

## 4. Discussion

CHPS is one of the few community-based primary care and prevention programmes in sub-Saharan Africa that has been shaped through pragmatic experimental research conducted within the delivery context. The early studies from the Navrongo Experiment show significant reductions in child mortality and improvements in uptake of family planning. While the studies in our review highlight many of the challenges in the adoption of the approach across all locations and in implementation, where CHPS was implemented according to the “15 steps,” delivery was more likely to be successful.

So why is it so challenging to scale-up what is evidently a successful approach? The literature on scale-up highlights the need for both vertical scale-up i.e., institutionalization, and horizontal scale-up i.e., increased coverage (91). Despite the initial skepticism of senior health advisers in the Ministry following the signing of the Bamako Initiative in 1989, the evidence from the Navrongo Experiment convinced health leaders to turn the approach into national policy and so the process of institutionalization, or vertical scale-up began.

Vertical scale-up has been identified as a pre-requisite for increasing horizontal scale-up (92). A review of studies reporting processes of scale-up by Milat et al. (93) has identified a number of factors which are frequently associated with success. Interestingly, many of these appear to have been present within the CHPS scale-up process, including systematic use of relevant evidence, strong leadership within the health sector and a well-defined scale-up strategy. The launch of CHPS as a national policy in 1999, and several subsequent reviews and revisions of the policy and “Implementation Guideline,” the most recent of which took place in 2016, make use of monitoring and research to strengthen implementation. The development of CHPS training with the clarity of the 15 steps and the six milestones are in-line with scale-up frameworks which emphasize the importance of simplifying and clarifying the intervention (91).

The use of costing and economic modeling of intervention approaches to inform policy and resource allocation was recommended by Milat et al. (93) as a strategy for successful scale-up. However, it is notable that the evidence base does not tend to take this into consideration. Only one study, Ferrer et al. (48), looked at cost-effectiveness of CHPS compared to integrated community case management (iCCM) to treat three infectious diseases. None of the studies took a broader approach to assessing costs and effectiveness across the range of primary care outcomes that CHPS is designed to address. Several of the qualitative studies highlighted the lack of resources within Ghana's health sector as a major limitation to the successful delivery of CHPS (35, 85). The decrease in donor funding due to donor transitions has compounded the funding challenges facing the CHPS programme. Increasingly, this means that budgetary allocations to primary health care and the CHPS programme from the Government of Ghana are insufficient. With few countries on the continent meeting the target of 15% of government expenditure on healthcare as agreed in the Abuja Declaration of 2001 (3), these challenges are common. However, the lack of government funding makes CHPS increasingly reliant on internally generated funds from the NHIS, out-of-pocket expenditure and funds from vertical programs and projects. Each of these sources present significant challenges to a strong health system-led by primary health care, with out-of-pocket expenditure undermining accessibility and vertical programmes leading to a focus on specific diseases rather than the holistic needs of the patient (1).

Our findings highlight challenges with horizontal scale-up, or increased adoption (in the language of RE-AIM), in certain geographical contexts including remote rural areas and urban areas. The challenges of delivering primary health care in remote areas are well-covered in the literature, with poorly maintained infrastructure, and a lack of supervision and managerial leadership cited as leaving those working in primary health care demoralized and suffering from burn-out (3). CHPS research, monitoring and evaluation has traditionally focused on rural areas because of the perception that Ghana's major primary health care challenges were rural. However, Ghana has evolved from a country that was 40% urban when the Navrongo pilot was conducted in 1994-5. Current estimates suggest over 57% of the population are now living in urban areas (94), and with an estimated urban growth rate of 4.2%, the urban population is expected to reach 65% by 2030 (7).

Increasingly questions arise as to how to adapt and deliver primary health care systems developed for rural poor populations to urban poor populations. This has led to increasingly attention to urban primary care in research and policy (95, 96) with findings pointing to the value of exploring different approaches to structuring primary health care, including building linkages between the plethora of private, informal and NGO providers with the more limited public sector primary health care providers (97). Developing strong community engagement and integration of volunteers, which is a key feature of the CHPS model, is a particular challenge in urban contexts. Strategies tried elsewhere include moves to pay CHVs regular stipends, as recently agreed in Kenya (98) and implemented in informal settlements in Bangladesh through the Manoshi programme, where volunteers receive financial incentives for each pregnancy identified or woman that they accompany to a delivery center (99). The need to adapt CHPS to fit the fast-evolving urban setting highlights a tension between clearly specifying the programme—as typified by the 15 Steps—and being able to allow flexibility and adaptability.

### 4.1. Strengths and limitations

A strength of the review is the wide search strategy used to identify both published and gray literature. However, given the diverse actors—NGOs, INGOs, donors, and researchers—who have been involved with the CHPS programme since its inception, it is likely that some evaluations will have been missed. Our systematic use of the RE-AIM framework to categorize the qualitative studies and to structure our synthesized findings is a further strength of our review. The review team also acknowledged throughout the review process that the use of the RE-AIM framework was at times challenging as findings did not always fit neatly into the RE-AIM domains. In particular, aspects of the context were hard to capture within the RE-AIM framework and this may have undermined insights in our synthesis.

### 4.2. Lessons for policy and practice

The review highlights the need to identify the resources required to successfully implement CHPS within the different socio-economic and socio-cultural contexts of Ghana. Clearly, adequate resourcing and strategies to meet the financial requirements of the programme are urgently needed. With reducing donor funds, the role and functionality of NHIS and its contributions to CHPS are of fundamental importance.

While the clarity of the steps needed to establish CHPS has undoubtedly helped with scale up, flexibility and nimble responses are needed in the context of rapid urbanization, health security in the face of pandemics and the changing disease burden exhibited within different contexts. The challenges of chronic diseases such as hypertension and diabetes, poor mental health, tobacco, alcohol and substance abuse are especially rife within urban populations, thus health needs will differ from those in a more traditional CHPS setting, and thus require a different approach. Ensuring that CHPS is not pulled too far from its original focus on promotion and prevention is particularly crucial given the increasing prevalence of non-communicable diseases. The studies included that focus on the urban context highlight the need to challenge assumptions that urban populations are already well-served by primary care. The predominant use of private, often unregulated health services and the lack of prevention highlight the need for an urban-specific CHPS model.

Keeping true to the original focus on community engagement is key, however, creative thinking to respond to the changing types of communities we find in rapidly urbanizing cities is needed. This may involve linking with occupational community structures such as market-traders associations or savings groups that are active in poor urban neighborhoods in addition to engaging with traditional leaders. Careful consideration of how to incentivize engagement is required in the urban context where volunteer time has a high opportunity cost. Given the rich history of evidence-informed programme development that characterizes CHPS, it is hoped that further research focusing on strategies to address the financial, service provision and community engagement challenges will continue to inform and improve CHPS.

## 5. Conclusions

The CHPS programme is built on a sound body of evidence, and clear specification together with a conducive national policy environment has aided scale-up. The combination of community health nurses and volunteers, with significant community engagement has been found effective in reducing under five mortality, particularly for the poorest and least educated, increasing the use and acceptance of family planning and reducing the fertility rate. While it is clear that the CHPS strategy can work for these rural populations in improving these outcomes, effectiveness in urban contexts is yet to be established. A clear specification of CHPS and a conducive national policy environment has aided scale-up, with strong community engagement, adequate resourcing and motivation for community health workers proving key to successful implementation. However, challenges to implementation and adoption across Ghana remain, particularly in urban and remote rural areas where these aspects are hard to deliver. Strengthened health financing strategies, review of service provision in light of pandemics, prevalence of non-communicable diseases and adaptation to changing community contexts will be required for future successful delivery and scale-up of CHPS.

## Data availability statement

The original contributions presented in the study are included in the article/Supplementary material, further inquiries can be directed to the corresponding author.

## Author contributions

HE, MA-O, AG, AA, AA-O, DD, EA, KA-W, and IA developed the protocol and concept of the review. MA-O, HE, AA, AG, AA-O, DD, EA, and KA-W screened and extracted data from the included studies. HE, LW, AN, DA, and AG coded qualitative findings. HE and DA synthesized qualitative findings. AV and HE synthesized quantitative findings and conducted the overall synthesis of results. HE drafted the manuscript with support from NA. All authors read and approved the final manuscript.
