# Supplementary material for: Implementation of the Community-based Health Planning and Services (CHPS) in rural and urban Ghana: a history and systematic review of what works, for whom and why
Source: Front Public Health. 2023 Jun 26;11:1105495. doi: 10.3389/fpubh.2023.1105495 (PMC10332345; doi:10.3389/fpubh.2023.1105495)
Supplement: Supplementary file 1 [file Table_1.DOCX]

**Supplementary Tables**

Table S1: Search terms

| CHPS | community-based health planning and services |
| --- | --- |
|  | Community health/ communit* adj3 health |
|  | Community engagement |
|  | Community participation |
|  | Community health officer(s)/ CHO |
|  | Community health nurse(s)/ CHN |
|  | Community health volunteer(s)/ CHV |
|  | Community health team |
|  | CHPS compound(s) |
|  | Community health management committee |
|  | CHPS strategy |
|  | CHPS+/CHPS plus |
|  | CHPS zones |
| Ghana (all regions of CHPS implementation) include new regions | Greater Accra |
|  | Volta |
|  | Northern |
|  | Brong Ahafo |
|  | Central |
|  | Eastern |
|  | Upper East |
|  | Upper West |
|  | Ashanti |
|  | Western |
|  | North East |
|  | Svannah |
|  | Oti |
|  | Bono Weast |
|  | Ahafo |
|  | Western North |
| CHPS implementation | Mechanism(s) |
|  | Facilitator(s) |
|  | Barrier(s) |
|  | Gap(s) |
|  | Lesson(s) |
|  | Scale-up |
| CHPS health outcomes | Maternal and child survival |
|  | Family planning |
|  | Reduction in childhood mortality |
|  | Health promotion and prevention |
|  | Maternal and infant mortality |
|  | Non-communicable diseases: e.g. CVD, hypertension, stroke, diabetes |
|  | Infectious diseases: e.g. malaria, gastro-intestinal, HIV, TB |

Table S2: Inclusion and exclusion criteria

| *Inclusion criteria* | *Exclusion criteria* |
| --- | --- |
| 1. Studies reporting on CHPS services delivered in rural and urban settings from 1994 to 2020 2. Studies reporting all outcomes (health and health system) achieved by CHPS including improving equity in access to basic health services, 3. Studies reporting CHPS implementation and scale up in rural and urban communities 4. Studies reporting on CHPS staff and their perspectives on CHPS implementation 5. Studies on CHPS reporting primary data from the following study designs:    - Randomised controlled trials (RCTs) with randomisation at individual or cluster level,    - Quasi randomised study designs and cluster quasi RCTs, where participants are allocated by some means other than randomisation (e.g. on case number, date of birth etc.),    - Non-randomised intervention studies that are prospective and have a control group, including:  - Interrupted Time Series that clearly define intervention points and record at least three outcome measurement points before and after (or before and during) the intervention. - Controlled before and after studies, where data collection must be contemporaneous and groups comparable on baseline scores. - ‘Before and after’ studies that do not have a control group, before and after studies that do not follow individuals over time, but conduct more than one cross-sectional assessment. Cross-sectional studies at one point in time. The findings from these study design may provide useful information on the nature and context of the CHPS programme and the mechanisms that may support effectiveness.   - Qualitative studies of all designs including case study, phenomenology, ethnography, grounded theory and studies using action-research approaches. | 1. Single subject/participant designs, reviews, overviews, surveys, commentaries, and editorials 2. Thesis with empirical data that had been subsequently published elsewhere. |
